# Three-Dimensional Printing Guided for Double Meniscal Allograft Transplantation

**DOI:** 10.1016/j.eats.2025.103542

**Published:** 2025-04-10

**Authors:** Paula Andrea Sarmiento Riveros, Alejandro Jaramillo Quiceno, Rubén Darío Arias Pérez, Mateo Velásquez López, Miguel Vega Arango, Juan Camilo Mejía Henao

**Affiliations:** a Orthopedic and Traumatology Service Clinica del Campestre, Medellín, Colombia; b Orthopedic and Traumatology Service Salud Sura, Medellín, Colombia; c Trauma and Orthopaedic Surgery resident of Pontifical Bolivarian University, Medellín, Antioquia, Colombia; d Trauma and Orthopaedic Surgery, Pontifical Bolivarian University, Medellín, Colombia; e MSK, Diagnóstico Cedimed, Medellín-Colombia; f Surgery Service, Salud Sura, Medellín-Colombia

## Abstract

In complex cases of meniscus allograft transplantation, such as in double transplants, and when they are combined with ligament reconstructions, there is a high risk of tunnel overlap, which may compromise graft integrity and lead to treatment failure. Additionally, matching the meniscal allograft size to the patient’s knee anatomy is critical and challenging. To address these challenges, a preoperative protocol was developed using three-dimensional (3D) printing. First, tomography of the knee is used to make a 3D model of the proximal tibia. This model is printed in real size and is used for allograft selection. The surgical team uses the model to determine the drilling parameters of the tunnels for the double meniscus allograft transplantation and anterior cruciate ligament reconstruction, ensuring that no overlap occurs. Finally, the 3D model serves as a guide during surgery for accurate tunnel and graft placement. The 3D model provides a versatile tool for planning meniscal transplants, especially in revision surgeries or complex cases, improving preoperative visualization and surgical precision. This technique can ensure optimal graft selection, facilitate precise replication of the drilling parameters, and enhance surgical outcomes.

The meniscus is crucial in joint stability, load absorption, proprioception, and other functions. The medial and lateral menisci bear approximately 50% and 70% of the load in their respective compartments.^1^ Irreparable meniscal injuries behave similarly to meniscectomy, disrupting biological and biomechanical functions, generating symptoms of instability and pain, increasing tibiofemoral contact pressure, and accelerating the progression of osteoarthritis up to 5-fold.^1^^,^^2^

For young patients with meniscal deficiencies where unicompartmental knee arthroplasty is unsuitable, meniscal allograft transplantation (MAT) is a widely accepted treatment. Grafts, whether cryopreserved, fresh-frozen, or fresh, aim to restore biomechanics, reduce pain, and improve outcomes. Mid-term (5-10 years) survival rates are 85.8% for medial and 89.2% for lateral grafts, decreasing to 52.6% and 56.6% after 10 years. Failure rates are 10.9% at 5 years, 22.7% at 10 years, and 17.8% overall.^3^

Accurate graft sizing is crucial for MAT success. Incorrect sizing disrupts biomechanics, unevenly distributes joint forces, and accelerates graft failure. Undersized grafts increase tibiofemoral pressure, risking premature failure, while oversized grafts cause impingement, extrusion, and overloading, leading to failure.^4^^,^^5^

To improve outcomes, various techniques for measuring graft size have been developed. Traditional radiographic methods are based on measurements of tibial plateau width and meniscal height.^5^ These measurements are pivotal in determining the appropriate graft dimensions, although their accuracy can sometimes be limited by the 2-dimensional nature of radiographic images.^6^ The introduction of advanced imaging techniques, such as computed tomography (CT) and magnetic resonance imaging (MRI), has significantly enhanced the accuracy of graft sizing.^6^ CT imaging offers a 3-dimensional (3D) visualization of the tibial plateau, providing high-precision measurements within 2 to 5 mm of actual size, a substantial improvement over traditional methods.^7^^,^^8^ This heightened precision reduces the likelihood of graft size mismatches, which can otherwise compromise the surgical outcome.^8^

MRI, although commonly used, has yielded more variable results. Some studies suggest that MRI can sometimes underestimate the actual size of the meniscus, leading to potential complications if not adjusted accordingly.^6^ However, indirect sizing techniques that use 3D models of the tibial plateau have emerged as a more reliable method, surpassing the accuracy of previous radiographic approaches.^7^^,^^8^ These advancements highlight the importance of precise preoperative planning and modern imaging in optimizing MAT outcomes. This article discusses a combined double meniscal allograft and ACL reconstruction technique using a 3D proximal tibia model for allograft selection and preoperative planning.

## Surgical Technique

### Preoperative Planning

**Fig. 1:**
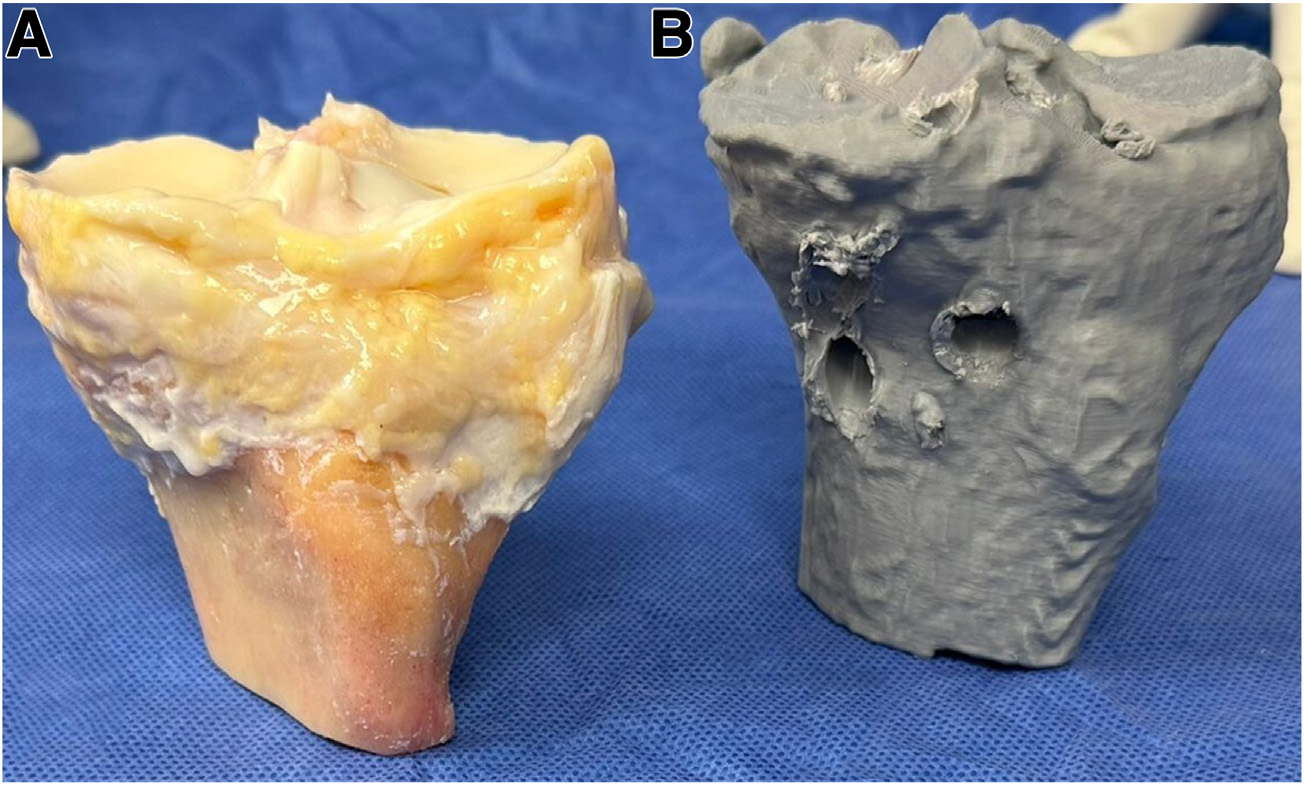
The 3D model is used to help select the most appropriate size allograft, visualization from an anteromedial perspective. (A) Fresh proximal tibia allograft with meniscus. (B) The three-dimensional life-size model of the proximal tibia.

**Fig. 2:**
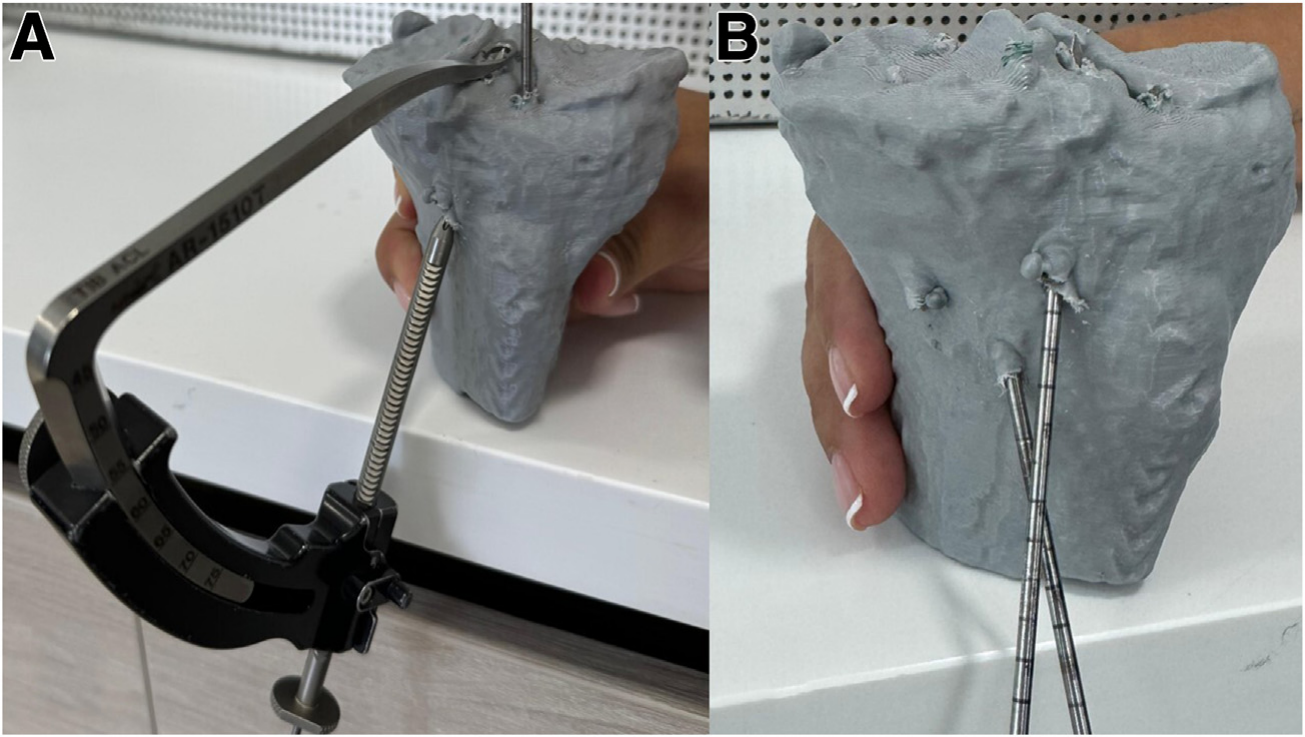
Preoperative planning, definition of tunnel parameters for guidewire angulation and exit of the tibial tunnel, visualization from an anteromedial perspective. (A) Tunnels for anterior cruciate ligament reconstruction and tunnel for the anterior root of the lateral meniscus. (B) The position of the different tunnels is checked to ensure that there is no convergence.

Using a 3D CT scan of the affected knee, a 3D digital model is obtained, which is printed in life-size using a resistant material (Hyper PLA filament). The 3D model is sent to the tissue bank to facilitate the appropriate selection of the meniscal allograft (Fig 1). Using the 3D model and nonsterile surgical instruments, the team carefully plans and creates the tunnels required for the bilateral meniscal transplant and ligament reconstruction. At this stage, the precise location and angles for the guide used to make the 5 tibial tunnels are determined, ensuring no tunnel overlaps (Fig 2). Finally, the 3D model is sterilized and used as a template in the operating room to ensure accurate tunnel positioning.

### Patient Positioning and Setup

**Fig. 3:**
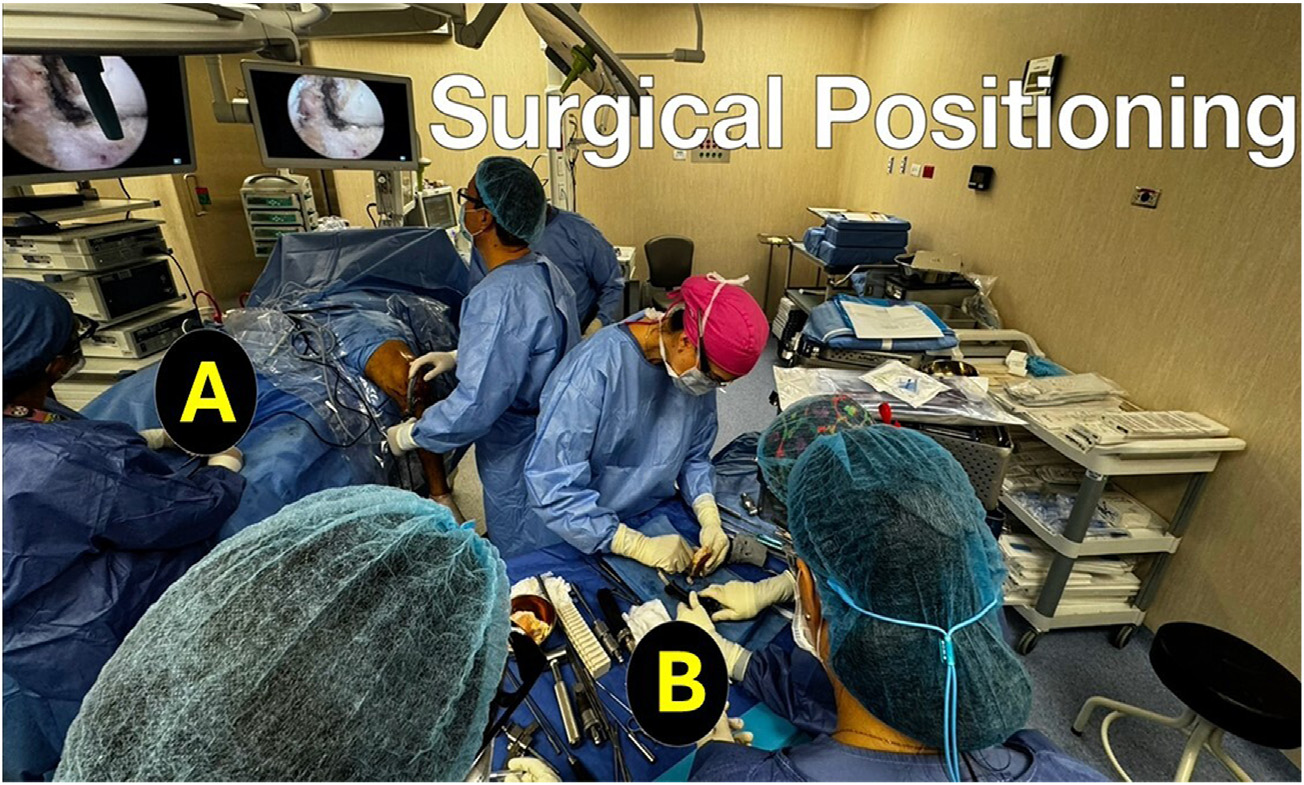
Two surgical teams work simultaneously. (A) One surgeon performs the arthroscopy. (B) The other surgeon is working on preparing the meniscal grafts.

**Fig. 4:**
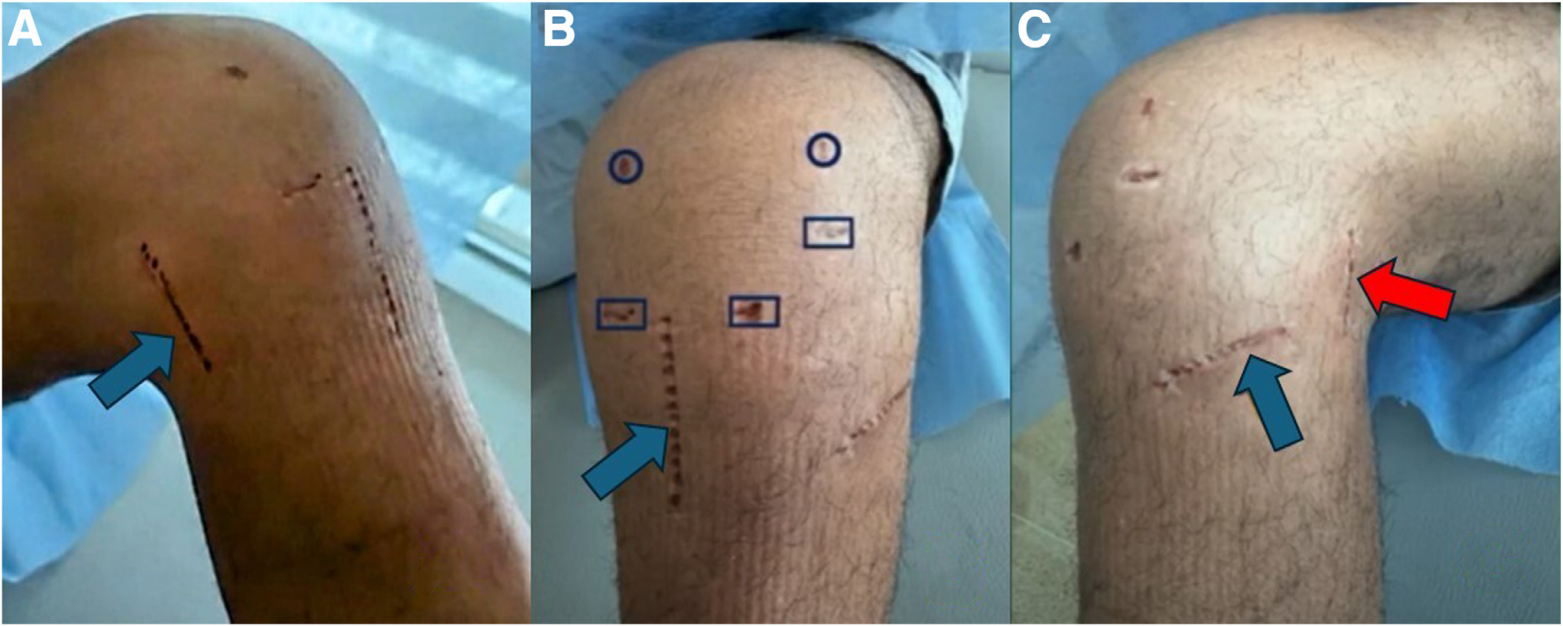
Portals and incisions are made on the left knee. (A) Anteromedial view, the blue arrow indicates the posteromedial approach. (B) Anterior view, conventional arthroscopic portals, and superior accessory portals for drilling anterior root tunnels. The blue arrow indicates the central incision for the anterior cruciate ligament reconstruction and the roots of the medial meniscus. (C) Anterolateral view, the blue arrow indicates the approach for the lateral meniscus, and the red arrow indicates the posterolateral approach.

Two teams work simultaneously; one prepares the graft, while the other works on the patient (Fig 3). The patient is placed in the supine position, and 5 portals are made: a superolateral, anteromedial, and transpatellar portal, and 2 more superior accessory portals to drill the tunnels of the anterior roots. Additionally, posteromedial or posterolateral approaches are performed. A central incision for the ACL reconstruction and medial anterior root is made, and a horizontal approach is used for the lateral anterior meniscus root (Fig 4).

### Graft Preparing

**Fig. 5:**
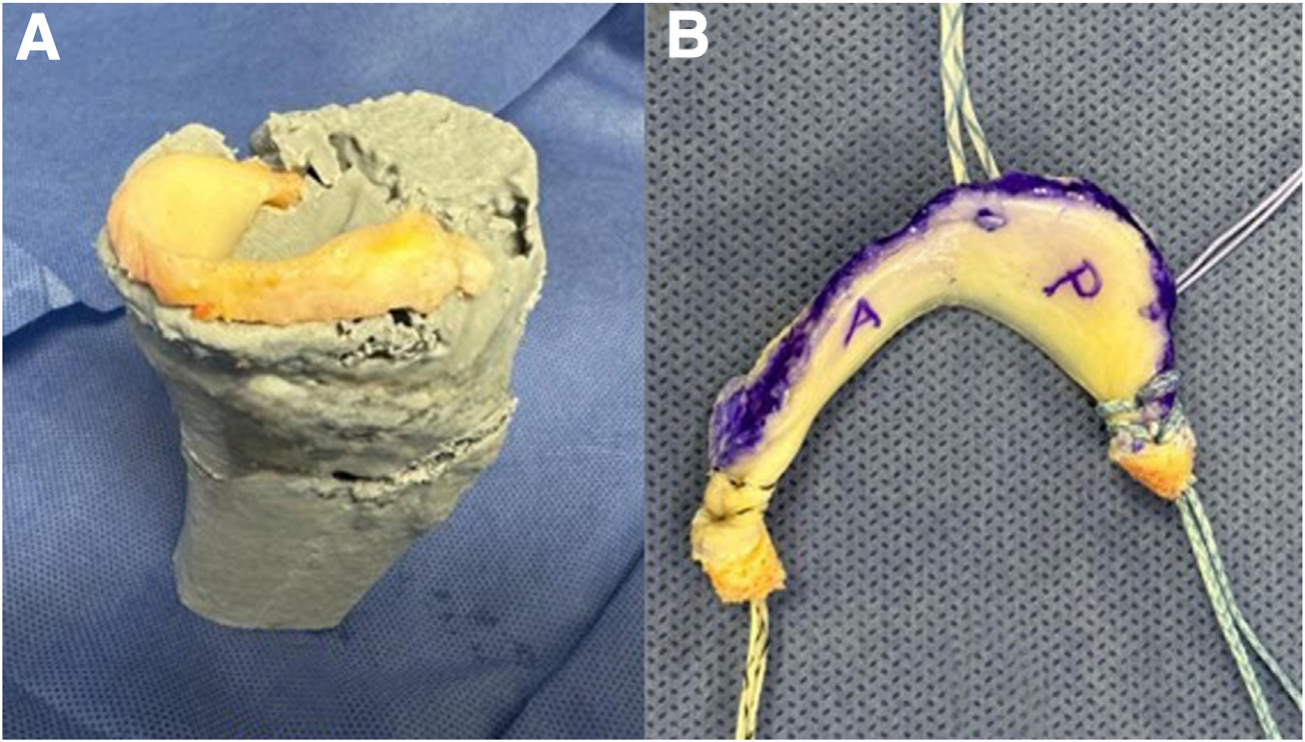
(A) One meniscal allograft is fitted to the recipient site with the assistance of the three-dimensional model. (B) The meniscal allograft is prepared with an 8-mm diameter bone plug attached to each root, while the rest of the meniscus is fixed using sutures. A marker is used to identify the periphery and the anterior and posterior horn of the meniscus. The sutures used in graft preparation are color-coded for easier identification of the different segments of the meniscus.

A fresh meniscal graft is used, and the transplantation is performed using a double-plug technique. The meniscal allograft is adjusted to the recipient site with the help of a 3D model, ensuring that it is anatomical, that there is no interference with the passage of the graft to the recipient bed, and that there is no extrusion (Fig 5). The graft is prepared with an 8-mm diameter bone plug attached to each root, allowing for secure bone-to-bone fixation of the meniscal roots, while the body of the meniscus is fixed using sutures. The sutures used in graft preparation are color-coded for easier identification of the different segments of the meniscus (Fig 5) (FiberWire, Arthrex, Naples, FL; Orthocord, Vicryl, and Ethibond Excel, Johnson & Johnson Medical, New Brunswick, NJ) (Table 1).

**Table 1:** Pearls and Pitfalls

Pearls	Pitfalls
▪ It is essential to identify and plan preoperatively all of the tunnels to be made in the tibia with the 3D model to reproduce them adequately without the tunnels converging. ▪ Use the 3D model to fit the meniscal allograft to the recipient site, ensuring there is no interference with the passage of the graft to the recipient bed. ▪ Use sutures as necessary to ensure the meniscal allograft is secure. ▪ The 3D model can also help assist MAT with associated surgeries, such as tibial osteotomy and ACL reconstruction at the same surgical time. ▪ Marking the edges of the meniscal allograft helps with orientation. ▪ The procedure should ideally be performed by 2 experienced surgical teams, which reduces time and errors.	▪ 3D segmentation before printing must be carried out in the most detailed manner possible since the anatomy of the model must be identical to the tibia to be treated. ▪ The preparation of the meniscal allograft should be performed by a surgeon with experience in this type of procedure to avoid failures. ▪ Using high-strength sutures of different colors when preparing the different portions of the meniscus allograft is essential to avoid confusion when positioning it. ▪ Other concomitant conditions such as instability and/or malalignment must be identified and treated to avoid poor outcomes.

3D, three dimensional; ACL, anterior cruciate ligament; MAT, meniscus allograft transplantation.

### Knee Arthroscopy

While the graft is being prepared, the patient team debrides the remaining meniscal tissue, leaving the capsule and part of the peripheral edge of the native meniscus intact.

### Tibial Tunnels for the Meniscal Allograft

**Fig. 6:**
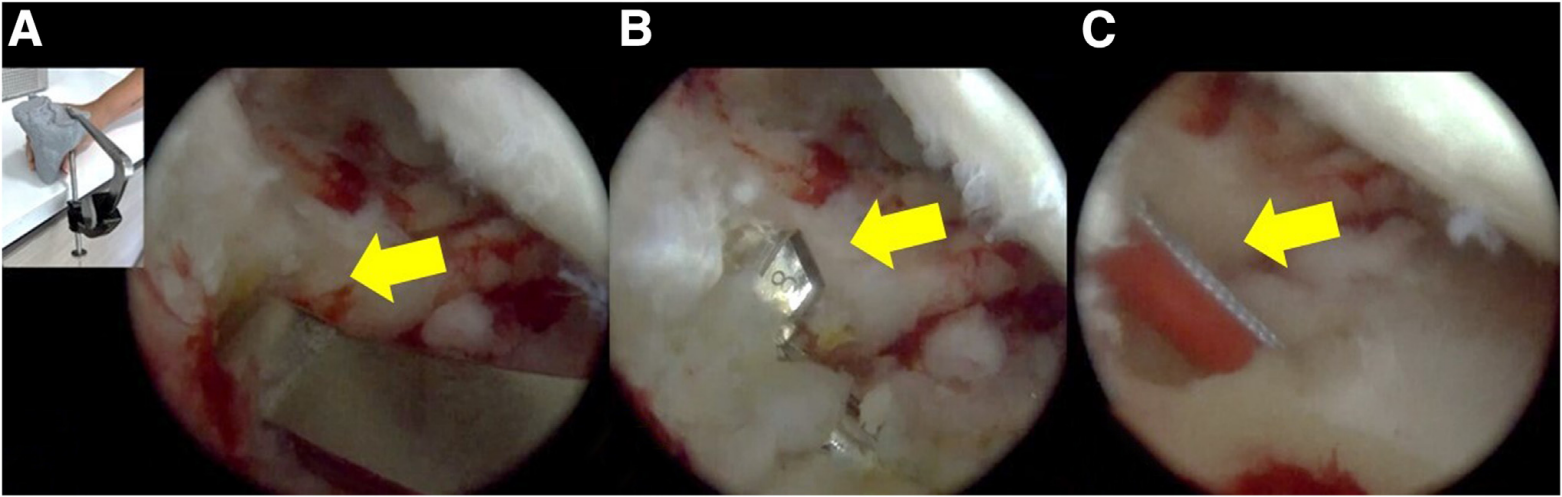
Arthroscopic view, preparation of the tunnel for the posterior root. (A) The posterior root is located following the previously planned angulations in the three-dimensional model to create the tibial tunnel; the yellow arrow points to the drilling guide. (B) A tunnel of 8-mm diameter and 10-mm depth is drilled using a retrograde drill; the yellow arrow points to the drill. (FlipCutter, Arthrex). (C) A suture is passed through the tunnel, to be used later as a traction suture; this is indicated with the yellow arrow (FiberStick, Arthrex).

**Fig. 7:**
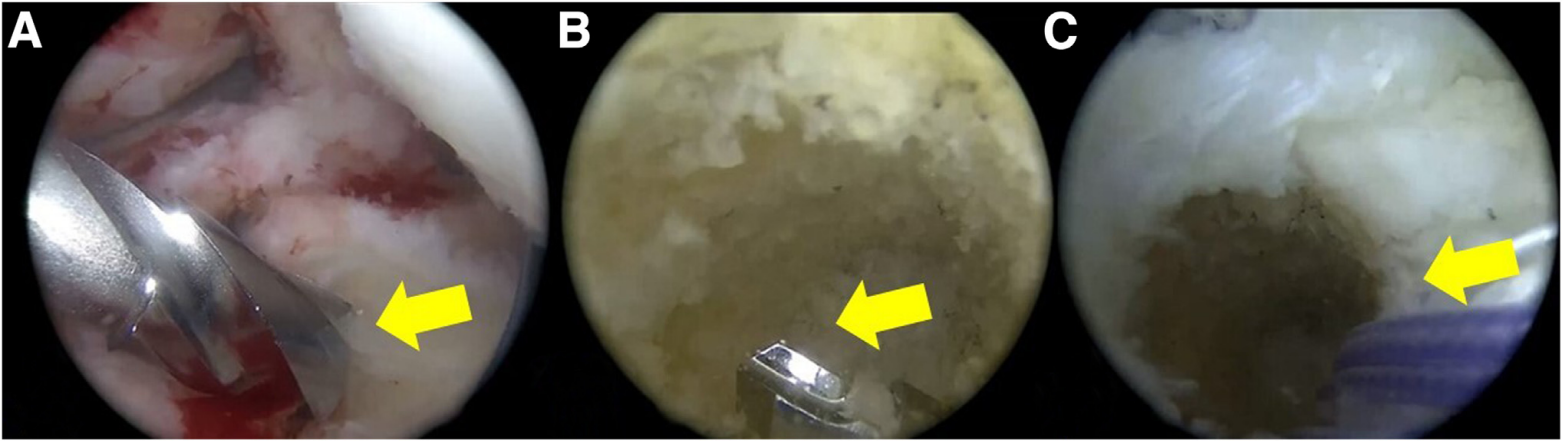
Arthroscopic view, preparation of the tunnel for the anterior root. (A) Using the superior accessory portals, the anterior root insertion site is located, and a tunnel of 8-mm diameter and 10-mm depth is drilled; the yellow arrow points to the drill bit. (B) Through this tunnel, another tunnel is made to allow the passage of sutures, connecting it to the anterior region of the tibia; the yellow arrow points to the drill bit in the new tunnel. (C) A suture is looped through the tunnel, to be used later as a traction suture; the yellow arrow points to the suture.

The meniscus insertion sites are exposed, and tibial tunnels are drilled, starting with the posterior root tunnel of the lateral meniscus, following the angulations previously planned on the 3D model for meniscus transplantation. For the posterior root of the meniscus, a retrograde tunnel of 8 mm in diameter and 10 mm in length is drilled (FlipCutter, Arthrex) (Fig 6). For the anterior root, superomedial and superolateral accessory portals are used to create a tunnel with an 8-mm drill with a depth of 10 mm. Using a C-guide, this tunnel is connected to the anterior region of the tibia, and a suture is passed through the tunnel (FiberStick, Arthrex) (Fig 7, Video 1).

### Meniscal Allograft Fixation

**Fig. 8:**
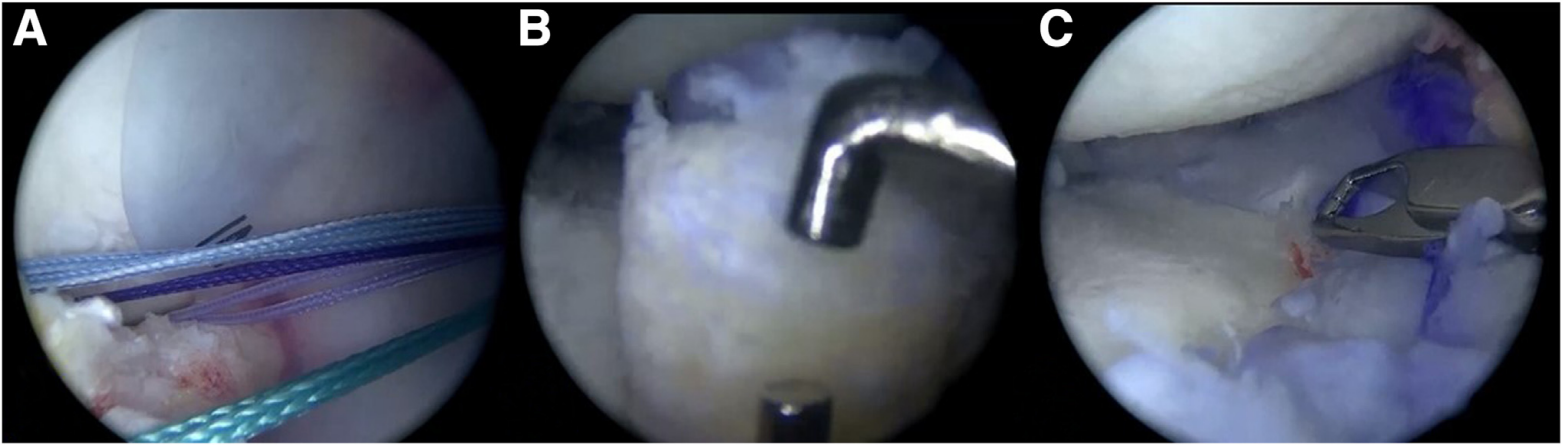
Arthroscopic view, passage of the meniscal allograft. (A) The meniscal allografts are inserted and positioned using a cannula in the recipient site. The different colored sutures used for traction are evident. (B) Insertion of the bone plug of the anterior root using an arthroscopic retriever. (C) The meniscal graft is finished positioning with the help of the arthroscopic forceps while the sutures are being pulled.

**Fig. 9:**
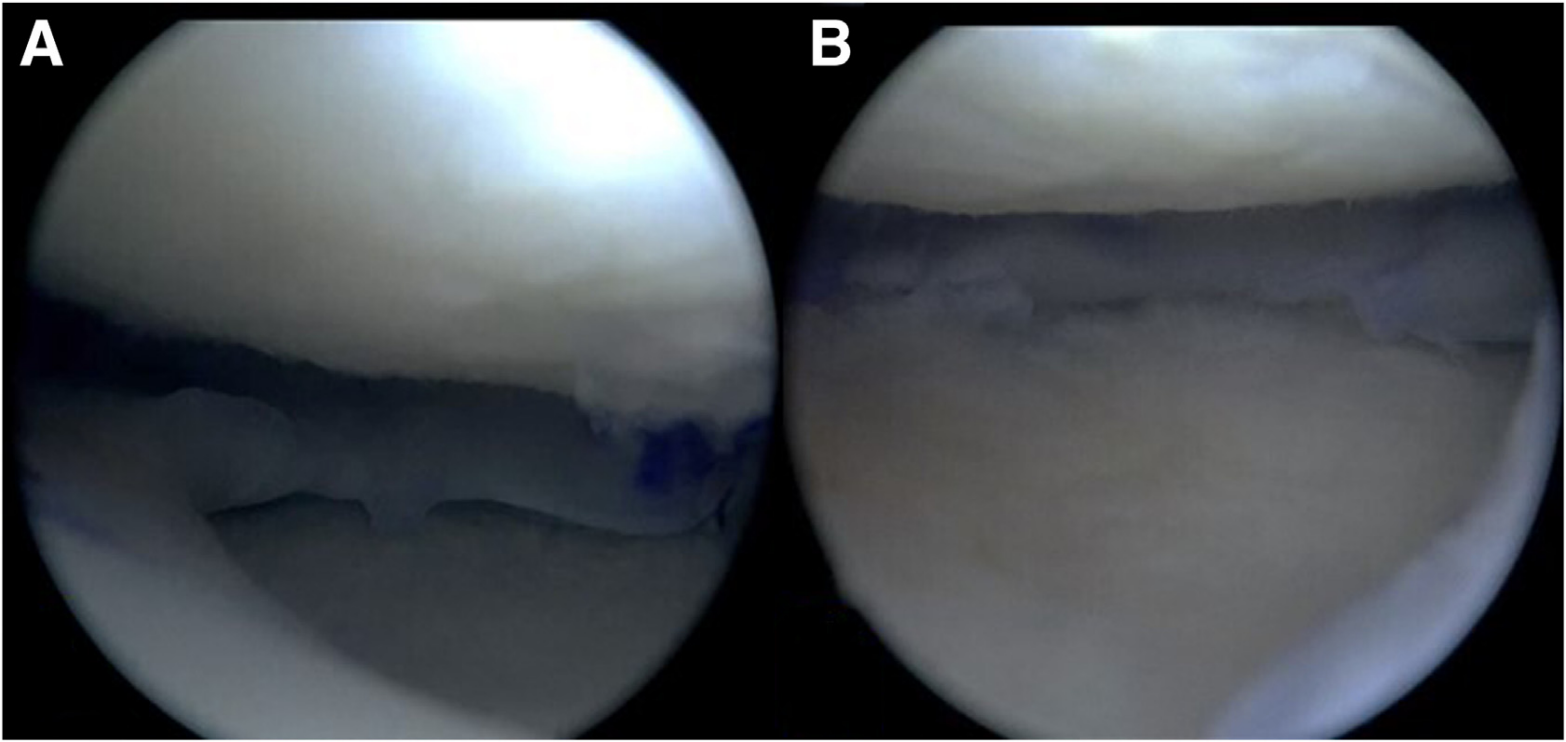
Arthroscopic view, positioning of the meniscal allografts in their recipient bed. (A) Medial meniscus. (B) Lateral meniscus.

Using a cannula (PassPort, Arthrex) (Fig 8), the meniscal allograft is inserted and positioned, achieving the planned alignment with the 3D-printed model. Posterior root fixation is performed using a pull-out technique and is subsequently fixed to the tibia using a suture anchor (SwiveLock 4.75, Arthrex). The anterior root is fixed using the same method. The rest of the meniscus is then sutured to the capsule using an inside-out technique for the body and anterior horn (Protector Meniscus Suturing Set, Arthrex). All-inside sutures are used to reinforce the posterior horn fixation (FiberStitch, Arthrex) (Table 1). First, complete fixation of the entire lateral MAT is performed, and then the same steps are repeated to fix the medial MAT (Fig 9). The medial collateral ligament pie-crusting technique is made for the medial compartment.

### Associated Procedures

**Fig. 10:**
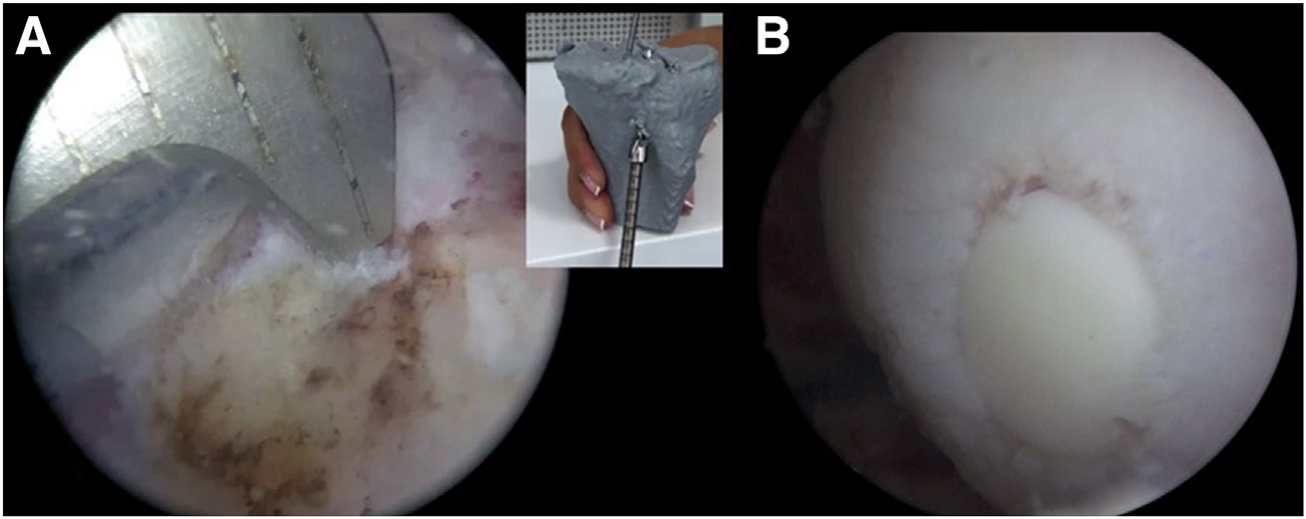
Performing concomitant procedures. (A) Following the previously planned angulations in the 3D model to create the tibial tunnels, the tibial insertion site of the ACL is located. (B) View of the osteochondral allograft transplantation in the lateral femoral condyle.

The tunnels for ACL reconstruction are made in the femur in position 70-30, and in the tibia, the tunnel is made considering the planning of the 3D model to avoid convergence between the tunnels (Table 2). After that, ACL graft placement follows, with fixation to the femur using an adjustable button (RigidLoop, Johnson & Johnson Medical) and to the tibia with an interference screw (Milagro Advance, Johnson & Johnson Medical). Finally, an osteochondral allograft transplant is performed on the lateral femoral condyle (COR, Johnson & Johnson Medical) (Fig 10, Video 1).

**Table 2:** Advantages and Disadvantages

Advantages	Disadvantages
▪ Graft selection is performed using a 3D life-size model of the proximal tibia, which may allow for a better choice of donor tissue. ▪ Meniscus selection and fixation based on the 3D model could help reduce the risk of extrusion. ▪ Planning surgery with a 3D model avoids overlapping tunnels in complex cases of MAT. ▪ Careful preoperative planning, with a precise definition of tunnel parameters for guide angulation and tibial tunnel exit, significantly reduces surgical time. ▪ The 3D model acts as a physical guide for the precise placement of tunnels and grafts during surgery, improving outcomes. ▪ The 3D model allows optimal anatomical adaptability of the grafts. ▪ This approach is especially valuable in revision surgeries, where previous tibial tunnels already exist, making it an innovative tool to manage complex cases requiring additional precision.	▪ The 3D printer to make the model adds cost and requires access to specialized technology, which may not be widely available. ▪ Although surgical time can be reduced, the preoperative phase requires more time and resources. ▪ Access to non-sterile instruments is required to create the tunnels in the 3D model.

3D, three dimensional; MAT, meniscus allograft transplantation.

## Discussion

This technique incorporates 3D life-size printing into the preoperative planning and execution of MAT. A 3D-printed model of the patient’s proximal tibia, generated from a CT scan, enables a more precise selection of donor tissue. Before transplantation, the dynamic matching process between donor and recipient facilitates more accurate tunnel placement and helps anticipate potential complications. It addresses one of the key factors influencing the long-term success of MAT. Additionally, the 3D model serves as a guide both preoperatively and intraoperatively,^9^ minimizing the risk of tunnel overlap—a complicated issue in combined procedures, such as double MAT and anterior cruciate ligament reconstruction. This method enhances surgical accuracy and reduces operative time, providing a detailed understanding of the recipient’s anatomy, potentially lowering the risk of graft extrusion and improving overall patient outcomes. However, the requirement for a 3D printer to make the model increases costs and necessitates access to specialized technology, which may not be widely available.

Several surgical techniques are available for meniscal transplantation, chosen based on surgeon preference and patient factors.^5^ Common methods include the suture-only, bone plug, and bone bridge techniques. The double-plug method, which transplants the meniscal graft with a bone block, provides stable fixation, reducing graft extrusion and improving outcomes. The soft tissue technique, using sutures without bone, is less invasive but may increase extrusion risk. Bone plug fixation has lower failure and reoperation rates compared to suture-only and bone bridge methods.^4^^,^^5^

Although less frequently reported, arthroscopic bicompartmental meniscal transplantation has been described in young patients with meniscal injuries, well-aligned joints, and no significant ligamentous laxity. When ligamentous instability is present, it can be corrected during the same surgical stage.^10^ Short-term results for bicompartmental MAT have been positive, suggesting that this technique may be a viable alternative for managing irreparable bimeniscal injuries.

Life-size 3D printing in MAT enhances surgical precision by enabling accurate donor selection and precise tunnel placement using patient-specific CT-derived models. It reduces risks like tunnel overlap, improves planning and execution, and shortens surgical times. As this technology becomes more accessible, its adoption is expected to grow.

## Disclosures

All authors (A.J.Q., P.A,S.R., R.D.A.P., M.V.L., M.V.A., J.C.M.H.) declare that they have no known competing financial interests or personal relationships that could have appeared to influence the work reported in this paper.

## Supplementary Data

Video 1 Description of the surgical technique: This video shows a surgical technique using three-dimensional (3D) printing to enhance precision in double meniscus allograft transplantation (MAT), especially in complex cases involving anterior cruciate ligament (ACL) reconstruction. A preoperative CT scan generates a life-size 3D-printed model of the proximal tibia, allowing for optimal allograft selection and precise planning of drilling parameters to prevent tunnel overlap. During surgery, the model serves as a reference to ensure accurate tunnel placement and graft positioning, improving surgical precision. This approach enhances visualization, reduces intraoperative risks, and could increase the success rate of meniscal transplants.
